# The Speed of Range Shifts in Fragmented Landscapes

**DOI:** 10.1371/journal.pone.0047141

**Published:** 2012-10-17

**Authors:** Jenny A. Hodgson, Chris D. Thomas, Calvin Dytham, Justin M. J. Travis, Stephen J. Cornell

**Affiliations:** 1 Department of Biology, University of York, York, United Kingdom; 2 Institute of Biological and Environmental Sciences, University of Aberdeen, Aberdeen, United Kingdom; 3 School of Biology, University of Leeds, Leeds, United Kingdom; University of California, Berkeley, United States of America

## Abstract

Species may be driven extinct by climate change, unless their populations are able to shift fast enough to track regions of suitable climate. Shifting will be faster as the proportion of suitable habitat in the landscape increases. However, it is not known how the spatial arrangement of habitat will affect the speed of range advance, especially when habitat is scarce, as is the case for many specialist species. We develop methods for calculating the speed of advance that are appropriate for highly fragmented, stochastic systems. We reveal that spatial aggregation of habitat tends to reduce the speed of advance throughout a wide range of species parameters: different dispersal distances and dispersal kernel shapes, and high and low extinction probabilities. In contrast, aggregation increases the steady-state proportion of habitat that is occupied (without climate change). Nonetheless, we find that it is possible to achieve both rapid advance and relatively high patch occupancy when the habitat has a “channeled” pattern, resembling corridors or chains of stepping stones. We adapt techniques from electrical circuit theory to predict the rate of advance efficiently for complex, realistic landscape patterns, whereas the rate cannot be predicted by any simple statistic of aggregation or fragmentation. Conservationists are already advocating corridors and stepping stones as important conservation tools under climate change, but they are vaguely defined and have so far lacked a convincing basis in fundamental population biology. Our work shows how to discriminate properties of a landscape's spatial pattern that affect the speed of colonization (including, but not limited to, patterns like corridors and chains of stepping stones), and properties that affect a species' probability of persistence once established. We can therefore point the way to better land use planning approaches, which will provide functional habitat linkages and also maintain local population viability.

## Introduction

There is a major concern that climate change and land use change could interact to cause the extinction of many species [1], [2], [3], [4], [5], [6]. Although many species are responding to climate change by shifts in their geographic range [7], [8], successful shifting depends on the availability of suitable habitat in regions of newly suitable climate [2], [4], [5], [9]. Conservationists urgently need to find out whether and how they can facilitate range shifts. It is fairly clear that the overall amount of habitat will be a major factor determining the speed of advance into newly suitable landscapes [2], [6], [10], [11]. However, there is no adequate theory to understand how habitat spatial arrangement affects the speed of advance, for a given total amount of habitat. Most previous studies of habitat spatial arrangement and fragmentation have been focused on minimizing the extinction risk of existing populations. In the absence of climate change, species generally have the best population viability in landscapes where habitat is spatially aggregated [12], [13], [14], [15]. During a process of range shifting, persistence is still important - subpopulations must still be able to persist for certain amount of time, but they must also have the capacity to found new populations before their climate window shifts away [5], [16]. Some theory using deterministic models suggests that aggregating habitat into larger clusters speeds advance [17], [18], [19], but the assumptions of these travelling wave models will break down in highly fragmented landscapes. Larger clusters usually means larger gaps between clusters, and this can obviously prevent range shifting if the species has a fixed maximum dispersal distance [20], [21]. The more realistic case, however, is when dispersal is probabilistic and rare, but not impossible, at long distances [22]. Some simulation case studies have observed faster advance with reduced levels of aggregation [2], [23], but have not explored the underlying reason for this. Here, we use a metapopulation framework to develop the first comprehensive analysis of how habitat arrangement affects the speed of range advance through fragmented landscapes.

## Materials and Methods

### Markov system analysis

We first developed an exact solution for the speed of advance, which can be computed for small numbers of patches. We use the fact that a metapopulation with N patches behaves as a Markov system with 2^N^ states. We assume an “initial state” with one patch occupied (henceforth referred to as the origin patch) and all others unoccupied. We define the speed of advance as the probability that a distant, “target patch” is ever colonized divided by the mean time taken until it is colonized (if it is colonized). The successful colonization of the target may happen via any number of intermediate states, as long as the “absorbing state” of extinction is not reached first. The calculation is made simpler by the fact that we can lump together all states where the target patch is occupied, and treat them as an alternative absorbing state, henceforth referred to as the “target state”.

The probabilities of transition between states are determined by combining the probabilities of individual patch transitions: each patch can go extinct with probability *μ* (we do not include a rescue effect) and each occupied patch can colonize an empty patch so that the colonization probability of an empty patch is 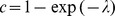 where 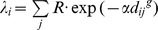, *j* indexes the occupied patches and *d_ij_* is the distance between patches *i* and *j*. The parameter *g* changes the shape of the dispersal kernel, with *g*<1 making it “fat-tailed”, with a higher proportion of long distance dispersal. The parameter *α* changes the mean dispersal distance for a given *g*. The parameter *R* changes the rate of production of propagules by an occupied patch.

The probability of ending up in the target state rather than extinction, given that the system starts in the initial state, that is with the origin patch occupied and all other patches unoccupied, is given by
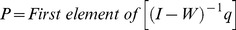
where *I* is the identity matrix, *W* is a matrix of transition probabilities between all non-absorbing states and *q* is a vector of probabilities of reaching the target state directly from each non-absorbing state. Conditional on not going extinct, the mean time to reach the target state is given by
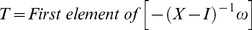
where *X* is *W* normalized to exclude the probability of extinction, and *ω* is a vector of ones. We combine these two quantities to give “speed” as P/T.

We evaluated speed for systems of between 1 and 8 patches between the source and the target, varying the colonization and extinction parameters and the spatial locations of patches. For more than one “stepping stone” patch, we did not investigate all possible spatial arrangements, but added stepping stones iteratively, each time choosing the location that gave the greatest increase in speed. For comparison we then calculated speeds given a number of idealized patterns, including a regularly-spaced chain of patches.

### Analysis with larger landscapes

We extended our analysis to consider a more realistic scenario where a species is found in one large landscape (of hundreds of patches), and has to extend its range into an adjacent, newly climatically suitable landscape. We found the speed of advance by simulating the metapopulation dynamics, because the exact calculation of speed was not feasible for such large numbers of patches (see “simulation” section below). We wanted to investigate the interplay between the probability of the chains of colonization events which are needed for the species' range advance, and the spatial clustering of habitat which is known to increase metapopulation viability. To this end, we investigated three families of spatial habitat pattern (see “landscape generation” section below), while keeping the total amount of habitat constant. We also aimed to test the performance of several summary metrics that might be efficient approximations to the speed of advance, and that could be used in conservation planning (see “landscape summary metrics” section below).

### Landscape generation

**Figure 1:**
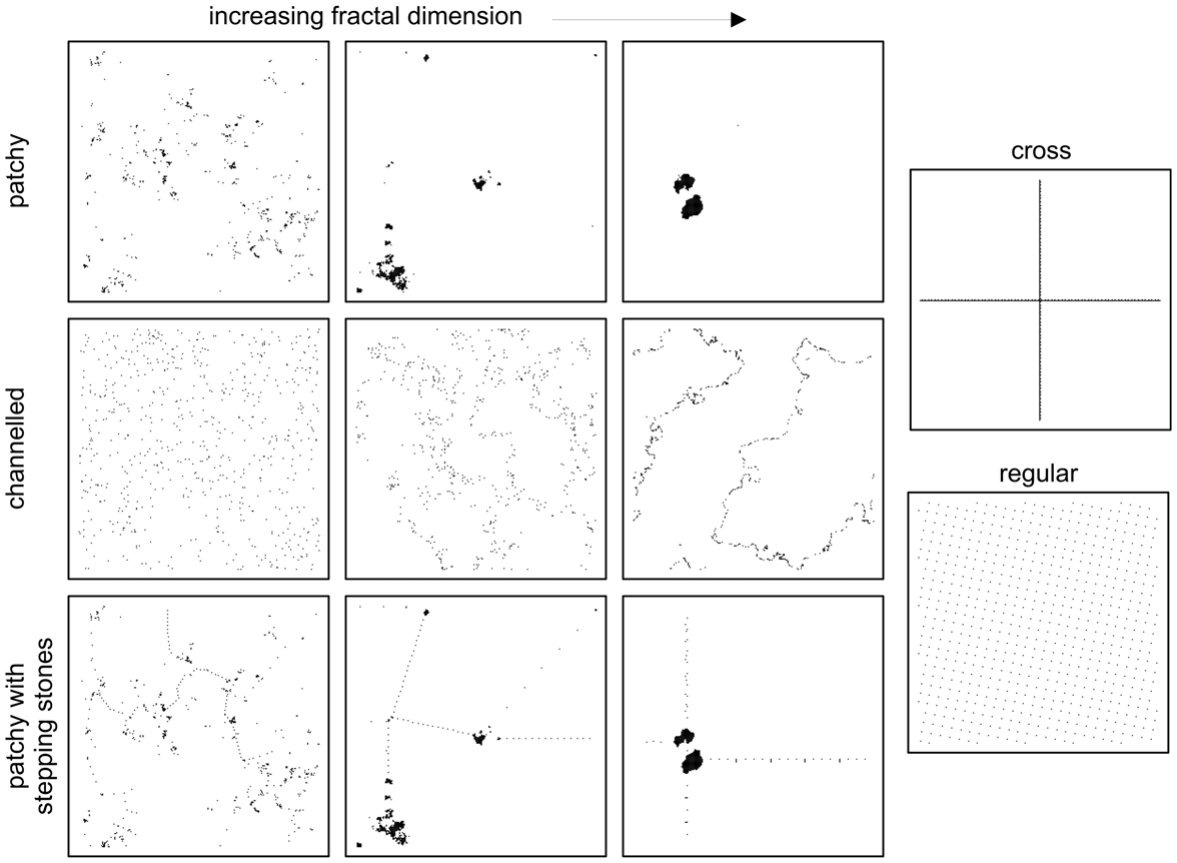
Examples of the landscape patterns used for simulations of range advance. Nine example landscapes generated from fractals are shown, along with the idealized “regular” and “cross” landscapes. All landscapes have the same overall amount of habitat: 1%. In total we used 10 randomly generated fractals for each of 11 levels of fractal dimension. The patchy landscapes consist of clusters of varying sizes, reminiscent of the pattern of many fragmented natural habitats. The channeled landscapes are the negative image of the patchy landscapes (i.e. they are the gaps left between clusters of non-habitat). Similar patterns may exist in nature for habitats associated with rivers or with ecotones. The patchy landscapes with stepping stones represent the kind of pattern that could be achieved with deliberate habitat re-creation (0.9% pre-existing patches and 0.1% stepping stones along the multiple shortest paths, see methods).

We generated a wide variety of habitat spatial patterns using fractals as a basis, as well as two idealized patterns, a strictly regular spacing of suitable cells, and a giant cross with continuous east-west and north-south corridors (landscapes illustrated in Fig. 1). The “patchy” family of landscapes (Fig. 1 top row) is a commonly used model in fragmentation studies [24], consisting of clusters of varying sizes, reminiscent of the pattern of many fragmented natural habitats. The “channeled” landscapes (Fig. 1 middle row) are the negative image of the patchy landscapes (i.e. they are the gaps left between clusters of non-habitat) and have the interesting properties of approximately regular spacing along the “channels” but higher-than-random aggregation across the whole landscape. Similar patterns may exist in nature for habitats associated with rivers or with ecotones. The “patchy landscapes with stepping stones” (Fig. 1 bottom row) represent the kind of pattern that could be achieved with deliberate habitat re-creation. Their overall aggregation is almost as high as the equivalent patchy landscape, but their “shortest path” from one edge of the landscape to the other is much shorter.

Continuous two-dimensional surface fractals on a 256×256 grid were generated by the method of Chipperfield *et al.* [25]. For each family of spatial pattern we used 10 randomly generated fractals for each of 11 levels of fractal dimension. The continuous values were ranked and then either the top ranking 1% or the median 1% of cells (655 cells) were picked to produce habitat for patchy and channeled landscapes, respectively. We decided to use a very low proportion of habitat in the landscape because at this extreme the effects of different spatial arrangements are most pronounced.

To produce patchy landscapes with stepping stones, the top ranking 0.9% of cells were picked, and the resulting landscapes analyzed to determine the quickest path between each cell and each of the four edges of the landscape (the multiple shortest paths, see “landscape summary metrics” section below) for a negative exponential dispersal kernel with a mean distance of 10. We added 64 habitat cells as stepping stones along these paths so as to minimize the maximum step distance, and bring the amount of habitat up to 1%.

The giant cross landscape consisted of one completely full row and one completely full column of cells (totaling 511 cells) and the adjacent row and column evenly populated with the remaining cells to make up 655. The regular landscape was generated by overlaying a 10×10 lattice over the landscape, at an angle (11°) to avoid extra space at the edge because 256 does not divide by 10.

### Simulation

We implemented a simple stochastic patch occupancy model. The colonization probability of each patch *i* is 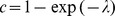 where 
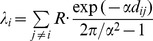
, *j* indexes the occupied patches and *d_ij_* is the distance between patches *i* and *j*. *R* can be interpreted as the number of emigrants leaving each occupied patch per time step (referred to as ‘fecundity’ for short), and 2/*α* as the mean dispersal distance. The extinction probability of each patch is *μ*(1−*c*), where the (1−c) term constitutes a rescue effect. We investigated mean dispersal distances between 1 and 10 cell units, *R* values of 10, 45 and 100 and *μ* values of 0.05, 0.2 and 0.4.

Each simulation started with a 200 time step “burn in” using one landscape tile (such as one of the landscapes in figure 1), initialized with 50% occupancy. Each habitat cell was treated as one patch (this assumption ensures that we are comparing like with like when we compare different spatial arrangements of habitat). Then, a duplicate, unoccupied landscape tile was appended either to the north or to the east, so that this empty landscape could be colonized from the existing one. The speed of advance was obtained from a linear regression of the most northerly (or easterly) occupied patch location vs. time (not including times after the population had reached the most distant patch). For initial exploration, every parameter combination was used to simulate expansion for 200 time steps (see Fig. S4). To further distinguish the landscapes with very slow expansion, we simulated expansion for up to 40,000 time steps for dispersal distances of 2, 4, and 8, with *R* = 100 and *μ* = 0.2 (see Figs S2 & S3).

### Landscape summary metrics

Because we observed in the Markov system analysis that the relative speed of different spatial arrangements was not much affected by the extinction probability (see results), we hypothesized that a simpler metric based only on the expected time to colonization between pairs of patches would be an efficient predictor of speed. This makes it possible to analyze systems with many more patches.

We represent the landscape as a weighted, undirected graph [26], or network, where each habitat patch is a node and the cost of each link is the expected time for a population to colonize one patch from another, assuming the first patch starts off occupied. Every node is connected to every other node. As in the simulations, each unit habitat cell is treated as a patch of equal quality. We do not lump contiguous cells into “patches” of different sizes because this would imply a change in the assumptions of the underlying model, making the speed of advance between some cells infinitely fast.

We decided to use the edges of the landscape (north and south or east and west) as nominal start and end points for our speed of advance metrics. The edges are represented in our network as special nodes which have a link to all the habitat cells but not to each other. The cost of links to the edges is based on the shortest straight-line distance between a cell and the edge. This is more-or-less equivalent to assuming that colonists are equally likely to come from anywhere along the edge. This assumption could be modified to suit particular conservation applications, using sources and targets relevant to species' predicted range shifts.

Having defined the network and the start and end points, the single shortest path is the chain of colonization events between start and end points which has the lowest summed time. The multiple shortest paths are the N shortest paths where each path is constrained to go through one of the N habitat cells. The maximum flow is calculated assuming the links in the network are drainage pipes and the capacity of each pipe is the reciprocal of the colonization time. The conductivity is calculated assuming the network is an electrical circuit and the resistance of each link is the colonization time. Shortest paths and maximum flow were calculated using standard algorithms in the R package igraph [27], [28]. Conductivity was calculated as
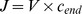
where
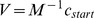
and *M* is a *N*×*N* matrix with elements
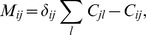
where *C* is the single step conductivity (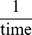) between any two points, *i* and *j* index the *N* cells, *l* indexes the *N* cells together with the start and end points, and δ*_ij_* is [0 if *i≠j* and 1 if *i* = *j*]. 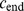 is a vector of 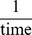 between each cell and the end point; 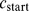 is a vector of 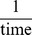 between each cell and the start point.

Versions of these metrics have been used before in modeling animal movement in heterogeneous landscapes [26], [29], but our approach to specifying the model (translating from ecology to mathematics) is different, in order to predict the population-level process of range advance. Previous applications have sought to model the literal trajectory of individual dispersers, given perceived differences in “costliness” of crossing different types of landcover. We are instead interested in modeling the advance of a population, where only successful dispersal to, and establishment in, a suitable habitat cell is regarded as advance, but the population can simultaneously occur in many patches (unlike an individual disperser). The expected time until colonization of one cell from another is calculated using a distance-based kernel. A straightforward extension to our approach would be to compute time to colonization from an explicit individual-based movement model instead.

Aggregation of the landscape was measured as the sum of colonization probabilities between all pairs of habitat cells, also known as metapopulation connectivity [30].

## Results

**Figure 2:**
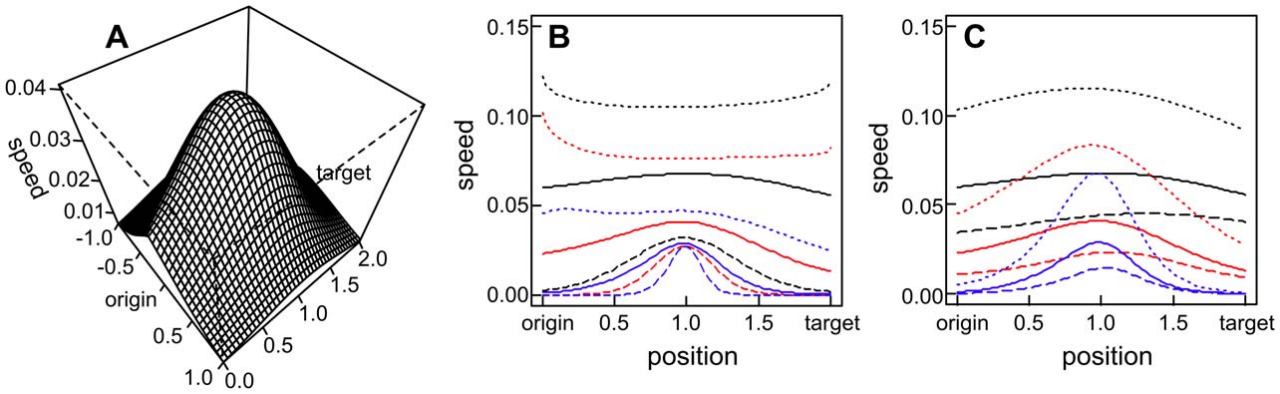
The value of a “stepping stone” of habitat as a function of its location. given a source patch at (0, 0) and target patch at (0, 2). Speed is defined as the probability of colonizing the target at all (not going extinct) divided by the mean time until the target is colonized. (**a**) Speed vs. location of the stepping stone shown in two dimensions (x, y) with one parameter set: colonization parameters *α* = 1, *g* = 1 and extinction probability *μ* = 0.2. (**b**) Speed vs. location of the stepping stone shown in one dimension, along the straight line between the source patch and the target patch, and the effect of varying the colonization kernel *α* = [1,2,4] shown with [black,red,blue] and *g* = [0.5,1,2] shown with [dotted,plain,dashed] lines where *μ* = 0.2. (**c**) the effect of varying the extinction probability *μ* = [0.05,0.2,0.4] shown with [dotted,plain,dashed] lines, with *α* = [1,2,4] shown with [black,red,blue] and *g* = 1.

Figure 2 gives exact results for a single stepping stone patch in-between the source patch and the target patch, showing how the speed of advance depends upon the stepping stone's location, and on the colonization and extinction parameters of the system. Recall that speed is defined as the probability of colonizing the target patch at all (not going extinct) divided by the mean time until the target is colonized. Over most of parameter space, the optimum location for the stepping stone is halfway between the source and the target (Fig. 2a–b). The cases where the optimum stepping stone location is closer to either the source or target occur when the speed is relatively fast, even without a stepping stone, and the maximum benefit provided by the stepping stone is low (Fig. 2b). The extinction risk of individual patches has surprisingly little effect on the optimal spatial arrangement (Fig. 2c), even though high extinction rates reduce the speed overall.

When adding up to eight patches to this simple metapopulation, a consistent result emerges that a regularly spaced chain is the best arrangement whenever the probability of colonization over the minimum spacing is low (Fig. S1). Again, we find that the extinction probability hardly affects the *relative* speed of different spatial arrangements (Fig. S1a stars vs squares).

The benefit of regular spacing can be appreciated through the following heuristic argument. The mean time to colonize one patch from another is proportional to exp(*αd^g^*) in the absence of any other patches (the lower the per-time-step probability of colonization, the higher the mean time until colonization; increasing *α* or *g* makes dispersal less likely at longer distances, *d*; *g*<1 makes a “fat-tailed” kernel, see methods). If a stepping stone is placed at a fraction *f* of the distance between the source and the target, the time to colonize the target via the stepping stone can be approximated as 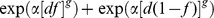. This function always takes its smallest value at *f* = 0.5, except in cases (*g* and α sufficiently small) when it is faster to colonize directly from the source to the target than to use the stepping stone at all. The benefit of having a centrally placed stepping stone as opposed to colonizing the target directly from the source (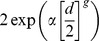 compared to 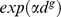) increases exponentially as *αd* increases (*αd* being the distance between source and target patch as a multiple of the species' typical dispersal distance). This explains the major patterns observed in figures 1 and S1: for parameters (species) where colonization is easy, stepping stones are not really needed and their locations do not matter much. For parameters (species) where colonization is difficult, stepping stones make an enormous difference and it is especially important for them to be regularly spaced. This is important for planning conservation for multiple species, because it means that there is not an inherent conflict between the needs of species with different dispersal abilities. If planners reduce the maximum distance between adjacent patches in a chain of stepping stones, they are helping the species that are most dispersal limited, without hindering the others (those with long distance dispersal for whom the spatial arrangement matters less [31]).

**Figure 3:**
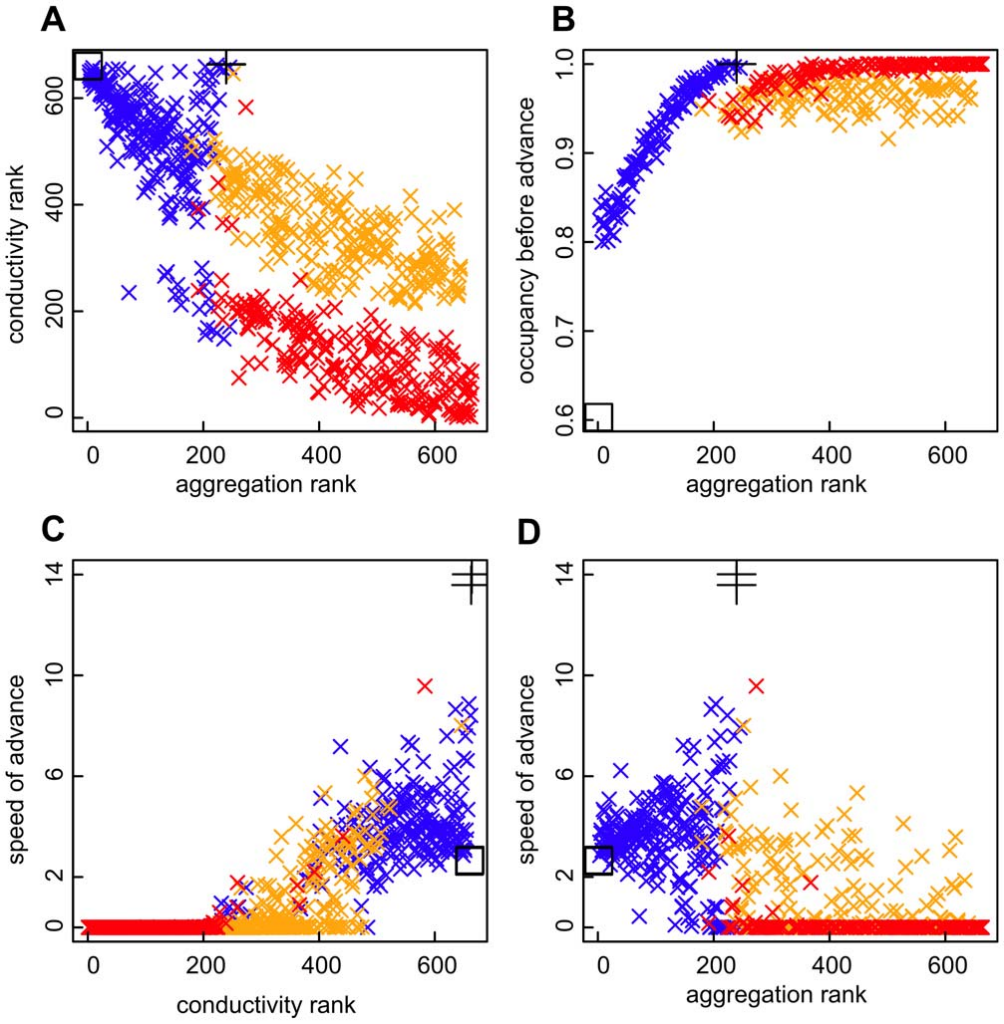
The trade-off between conductivity (good for speed of advance) and aggregation (good for steady-state occupancy). (**a**) Rank correlation between landscape metrics of conductivity and aggregation. Red points are from patchy landscapes, blue from channeled landscapes and orange from patchy landscapes with stepping stones. Aggregation increases with fractal dimension within each family of landscape (cf fig. 1). Large black cross represents the cross landscape and square represents the regular landscape. (**b**) The occupancy of landscapes in simulations before range advance started, at the end of the 200 time-step “burn-in”, against aggregation with symbols as in (**a**). (**c–d**) The speed of advance into the unoccupied landscape in cells per time step, against the conductivity (**c**) and aggregation (**d**) metrics, with symbols as in (**a**). Each point represents one simulation run. Metapopulation parameters were a mean dispersal distance of 8 cells, fecundity of 100 (propagules produced by an occupied cell) and per-cell extinction rate of 0.2.

In the simulations with hundreds of patches, landscapes with the highest spatial aggregation had the lowest rate of advance - often indistinguishable from zero (Fig. 3d). However, the converse was not true - a landscape of regularly placed patches did not give the fastest advance (Fig. 3c–d square symbol). In this two-dimensional landscape, channeling the habitat into one or a few chains creates faster routes for colonization, because it gives the benefit of regular spacing in the approximate direction of travel, as in the analytical model, and also avoids the low population persistence associated with patch isolation. The giant cross landscape exemplifies this property; it and a few other landscapes are characterized by high conductivity but intermediate aggregation (Fig. 3a), and always have the fastest speeds of advance (Fig. 3c–d). Landscapes with very low aggregation experience reduced occupancy because of patch isolation (Fig. 3b), as in any metapopulation model, but still allow range advance as long as the species does not go extinct (Fig. 3d).

The conductivity metric, derived from electric circuit theory, predicted the speed of advance better than other candidate metrics (see methods and Fig. S2). Conductivity can be tailored to different dispersal distances (Fig. S3) and fecundities (Fig. S3, S4). However, it does not explicitly incorporate extinction risk, and as a consequence it over-estimates speeds for the regular and near-random (lowest aggregation) landscapes (Fig. S3, S4). Without needing specialist software (using the matrix inversion functions built into R [27]), conductivity can be calculated quickly and easily for systems of up to a few thousand patches.

## Discussion

We have presented a new approach to calculate the speed that a species can shift into a fragmented landscape of habitat. We have developed both an exact calculation which can be evaluated for small numbers of patches, and an efficient approximation for large numbers of patches. The most important result is that spatial arrangements which maximize speed are quite different from the arrangements that would maximize persistence in a static climate. This has profound implications for conservation planning which aims to promote both persistence and shifting potential. It could also prompt a re-evaluation of some related questions in population dynamic theory.

### Advantages and limitations of our modeling approach

Our modeling approach includes the most fundamental population processes that allow a species to shift into a new landscape. There is a certain amount of habitat, in which reproduction is possible. Dispersal allows the colonization of new habitat; once a piece of habitat is colonized, it can produce new colonists after a certain time. We also include a risk of population extinction.

These fundamental processes are relevant, and can potentially be parameterized, for any species. However, some additional processes could be included within subsequent developments of the approach. For example, we did not include changes in population density within a patch, or density dependence of emigration. The patch occupancy model is thus an approximation where the time period between a patch being colonized and reaching carrying capacity is collapsed into a constant parameter: the model's time step, and subsequent fluctuations around carrying capacity can be ignored. The model also requires that space be divided into discrete patches, which can reasonably be assumed to be colonized and become extinct as units, and which maintain the same colonization and extinction parameters over time. Note that, although we have investigated spatial arrangement using habitat units of equal size and quality for simplicity, the modeling framework will work equally well with heterogeneous patches.

It is common in landscape ecology and metapopulation studies to treat any block of contiguous habitat as a single patch, however large it is. This is potentially problematic when investigating range expansion and invasion because it is not reasonable to assume that a very extensive patch will get colonized all at once. It is also common in such studies to assume that extinction risk decreases as a power law with patch area. This is argued to be a reasonable scaling for the chance of a catastrophic disturbance [32]. In our alternative approach, where each grid cell of habitat is considered independent, the effective extinction risk for a large block of habitat cells will be *lower* than that given by a power law. We are therefore using a rather optimistic assumption of the benefits of habitat aggregation, and thus we can be fairly confident that our result that aggregation generally reduces the speed of advance is robust.

Although we have investigated various shapes of dispersal kernel, we have not considered situations where colonization rates depend on properties of the intervening landscape, as well as the distance between patches. Our modeling framework is not tied to the use of dispersal kernels: any method could be used to calculate the probabilities of patch-to-patch colonization. One interesting scenario, which could lead to slightly different optimal habitat arrangements, is the existence of the “shadow effect” [33]. This happens when, during dispersal, individuals are likely to stop when they encounter any habitat, but more likely to continue dispersing if they do not encounter habitat. This effect could reduce the benefit of a stepping stone or corridor-like arrangement, especially an arrangement that was strictly linear. The magnitude of the effect would depend on the relative probabilities of stopping and continuing, the effective area encountered by the disperser (due to the tortuosity of its path and its perceptual range) and the distance between stepping stones. We would predict that the shadow effect would be less important in cases where the stepping stones are widely spaced relative to the species' typical dispersal distance, i.e. in the cases where we have already shown that the stepping stones are most advantageous to the speed of range advance.

Lastly, we have modeled the range expansion process as though a whole landscape instantaneously becomes climatically suitable, so that the species is free to move into it. In reality, the “window” of climatic suitability will constantly be shifting, and population growth rates will be lower and more variable near the edge of the window. It is straightforward to include more realistic climate change into a simulation model [20], [34], [35], [36], [37], but much more difficult to incorporate it into a single measure of speed, using either the Markov system approach or the conductivity approach. We still think that it is useful, though, to calculate speed of advance for a landscape of habitat, assuming it is all suitable, because this will give the maximum speed achievable. If this speed is much slower than the projected speed of climate change, then there is an obvious need for conservation action. If the speed is faster than the projected speed of climate change, then the species will be limited by its climate window and not by the spatial arrangement of habitat patches.

### Conclusions for population dynamic theory

Over recent decades, spatial ecological theory has played a major role in shaping landscape management for conservation [38]. However, the existing theory is not well suited to the current challenge of managing populations facing concurrent climate change and habitat loss. Population theory for conservation has been focused on creating (meta)populations that are resistant to extinction at a dynamic equilibrium, and generally recommends spatial patterns of habitats that are aggregated. Meanwhile, studies of invasive species have answered many theoretical questions about the speed of advance into newly suitable habitats, but have hardly explored the effects of habitat spatial arrangement, probably because the habitat of invasive species tends to be abundant [10]. Studies of disease epidemics could also give some relevant theoretical insights [39], but it is difficult to transfer insights because models often use spatially implicit host contact networks (i.e. where distance in the network does not map onto spatial separation). The susceptibility of the network to an epidemic is often approached theoretically by calculating *R_0_* [40], [41], but this approach implicitly assumes that any host is equally likely to be the original infected case. We have introduced a new conceptual framework for studying the speed of advance for a highly stochastic population, in terms of whether - and how quickly -the population colonizes a specific target location given a specific origin location. This is, to our knowledge, the first attempt to calculate “invasion” speed in landscapes where the assumptions of a diffusion approximation will not hold. We believe that our stochastic approach based on first passage times and probabilities could be an improvement over existing methods to explore the spread of an invasive species or a spatially transmitted disease where the spread is likely to start from one spatial extreme. Indeed, it could aid our understanding of how quickly populations will reach a new equilibrium after any major environmental change. It is a high priority for future modeling studies to use these methods where appropriate, because we have already shown that they can lead to different conclusions from deterministic models [18] in the case of how spatial aggregation of habitat affects the speed of advance.

### Conclusions for conservation

Our results highlight that the ability of a species to persist *in situ* and its ability to undertake wholesale range shifts are qualitatively different properties. Large clusters of habitat are still important to prevent the imminent extinction of many species, but this will not be sufficient to ensure their long-term survival if the environment becomes fundamentally unsuitable as a result of climate change.

Our key result is that spatial aggregation of habitat patches hinders the speed of advance, even though it increases the equilibrium patch occupancy. This result is very robust to the species parameters chosen. Furthermore, we find that it is possible to achieve both rapid advance and relatively high patch occupancy when the habitat has a “channeled” pattern, resembling corridors or chains of stepping stones. Importantly, the strongly channeled patterns give the fastest speeds for all species parameters, although the speeds for species with longer distance dispersal are less sensitive to the spatial arrangement of habitat [31], so there is not an inherent conflict between the needs of species with different dispersal abilities. The aggregate effect of many routes or channels cannot be summarized by any simple statistic of aggregation or fragmentation, but it can be quantified by our version of conductivity, measured between opposite edges of the landscape (or between alternative source and target locations relevant to climate change).

Although it is already recognized that an adjustment of conservation strategies is needed to facilitate climate-driven range shifts [5], [42], [43], [44], progress has been hampered by a lack of a common currency to define what is adequate or desirable. Conductivity provides one such currency, which can be compared to the speed of climate change, and is not reliant on human-defined classifications of a “corridor” or “stepping stone”. While our results in general lend some theoretical credence to landscape linkage projects that are already being designed, e.g. [45], the designs still need to be tested to see whether the amount, pattern and quality of habitat to be created will make sufficient difference to the conservation of target species. Our conductivity metric could be applied at a scale that was relevant to each target species, and could be used alongside established metrics of population viability to plan effective habitat networks for long-term conservation.

## Supporting Information

Figure S1 **The speed obtained with different arrangements of four stepping stones between an origin and a target** (a) for 9 different colonization kernels illustrated in (b). In both panels *g* = [0.5,1,2] is shown with [dotted,plain,dashed] lines and *α* = [1,2,4]×4/4*^g^* shown with [black,red,blue] (the correction 4/4*^g^* is to make the kernels more comparable in average height even though they have different shapes). In panel (a) two different extinction rates are also shown (stars: 0.2; squares: 0.4). The arrangement “found by iteration” is found by testing all locations on a 3 by 81 lattice between the source and the target and choosing the location that gives the highest speed for each new stepping stone in turn. This iterative patch addition is not a reliable way of finding the best arrangement for several patches, because early choices severely limit the options for subsequent arrangements.
(PDF)

Figure S2 **Speed of expansion in simulations versus four putative summary metrics of the landscape.** In all panels (a–d) the y axis is the rate of advance (cells/time step) of a simulated metapopulation across one of 332 landscapes (see Fig. 2) in one of 2 directions (east-west or south-north). Each point represents one simulation run. Red points are from patchy landscapes, blue from channeled landscapes and orange from patchy landscapes with stepping stones. Large black cross represents the cross landscape and square represents the regular landscape. The four different metrics (x axes) are explained in methods section “Landscape summary metrics”. The numbers at bottom-left indicate the number of points that have a speed indistinguishable from zero and a metric value beyond the scale of these plots (scales are chosen to show the more informative, non-zero rates clearly). Metapopulation parameters were a mean dispersal distance of 8 cells, fecundity of 100 and per-cell extinction rate of 0.2. Correlation with speed of advance r = 0.82 for conductivity, r = 0.65 for maximum flow, 0.54 for 1/shortest path and 0.50 for 1/multiple shortest path (variables not log-transformed for correlation calculation). Notice that all metrics predict the large difference between the patchy and channeled types of landscape, but the single and multiple shortest path metrics seriously overestimate the benefit that could be gained by adding a few stepping stones to the patchy landscapes.
(PDF)

Figure S3 **Speed of expansion in simulations for species with different dispersal distances.** In all panels the y axis is the rate of advance (cells/time step) of a simulated metapopulation across one of 332 landscapes (see Fig. 2) in one of 2 directions (east-west or south-north), and the x axis is the conductivity, whose value depends on the landscape arrangement and the dispersal kernel. Each point represents one simulation run. Red points are from patchy landscapes, blue from channeled landscapes and orange from patchy landscapes with stepping stones. Large black cross represents the cross landscape and square represents the regular landscape. Metapopulation parameters were fecundity *R* = 100 and per-cell extinction rate *μ* = 0.2. We observed that rate of advance is approximately equal to conductivity ×√*R*, plotted as a thick grey line. The black lines show the points where observed/predicted speed would be insufficient for the species to advance across the landscape in the maximum time allowed for the simulation (which was 10,000 time steps for a dispersal distance of 8, 20,000 for 4 and 40,000 for 2), and numbers denote the count of runs falling above or below these lines. Points with conductivity less than 10^−4^, all with speeds indistinguishable from zero, are not shown on the graph but are included in the counts.
(PDF)

Figure S4 **Speed of expansion in simulations for species with different fecundity and extinction rates** (*R* and *μ*). In all panels the y axis is the rate of advance (cells/time step) of a simulated metapopulation across one of 332 landscapes (see Fig. 2) in one of 2 directions (east-west or south-north), and the x axis is the conductivity. Each point represents one simulation run. Dispersal distance equals 8 for all panels. We observed that rate of advance is approximately equal to conductivity ×√*R*, plotted as a thick grey line. The black lines show the points where observed/predicted speed would be insufficient for the species to advance across the landscape in the maximum time allowed for the simulation (200 time steps). Red points are from runs where the starting patch occupancy (at the end of the 200 time-step burn-in) was less than 2/3, to show that these landscapes tended to have a lower speed than predicted from their conductivity.
(PDF)

Data S1 **Raw data for this study is made available here.**
(ZIP)
