# Supplementary material for: The Speed of Range Shifts in Fragmented Landscapes
Source: PLoS One. 2012 Oct 17;7(10):e47141. doi: 10.1371/journal.pone.0047141 (PMC3474837; doi:10.1371/journal.pone.0047141)
Supplement: Figure S1 — The speed obtained with different arrangements of four stepping stones between an origin and a target (a) for 9 different colonization kernels illustrated in (b). In both panels g = [0.5,1,2] is shown with [dotted,plain,dashed] lines and α = [1,2,4]×4/4g shown with [black,red,blue] (the correction 4/4g is to make the kernels more comparable in average height even though they have different shapes). In panel (a) two different extinction rates are also shown (stars: 0.2; squares: 0.4). The arrangement “found by iteration” is found by testing all locations on a 3 by 81 lattice between the source and the target and choosing the location that gives the highest speed for each new stepping stone in turn. This iterative patch addition is not a reliable way of finding the best arrangement for several patches, because early choices severely limit the options for subsequent arrangements. (PDF) [file pone.0047141.s001.pdf]

SUPPLEMENTARY MATERIAL FOR "THE SPEED OF RANGE SHIFTS IN FRAGMENTED LANDSCAPES"

Jenny A. Hodgson, Chris D. Thomas, Calvin Dytham, Justin M.J. Travis and Stephen J. Cornell 2012

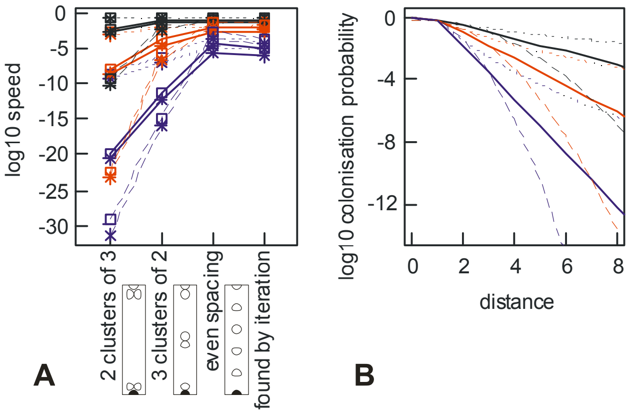

Figure S1: The speed obtained with different arrangements of four stepping stones between an origin and a target (a) for 9 different colonization kernels illustrated in (b). In both panels g = [0.5,1,2] is shown with [dotted,plain,dashed] lines and $\alpha = [1,2,4]\times 4/4^g$ shown with [black,red,blue] (the correction $4/4^g$ is to make the kernels more comparable in average height even though they have different shapes). In panel (a) two different extinction rates are also shown (stars: 0.2; squares: 0.4). The arrangement "found by iteration" is found by testing all locations on a 3 by 81 lattice between the source and the target and choosing the location that gives the highest speed for each new stepping stone in turn. This iterative patch addition is not a reliable way of finding the best arrangement for several patches, because early choices severely limit the options for subsequent arrangements.
