# Supplementary material for: The Speed of Range Shifts in Fragmented Landscapes
Source: PLoS One. 2012 Oct 17;7(10):e47141. doi: 10.1371/journal.pone.0047141 (PMC3474837; doi:10.1371/journal.pone.0047141)
Supplement: Figure S2 — Speed of expansion in simulations versus four putative summary metrics of the landscape. In all panels (a–d) the y axis is the rate of advance (cells/time step) of a simulated metapopulation across one of 332 landscapes (see Fig. 2) in one of 2 directions (east-west or south-north). Each point represents one simulation run. Red points are from patchy landscapes, blue from channeled landscapes and orange from patchy landscapes with stepping stones. Large black cross represents the cross landscape and square represents the regular landscape. The four different metrics (x axes) are explained in methods section “Landscape summary metrics”. The numbers at bottom-left indicate the number of points that have a speed indistinguishable from zero and a metric value beyond the scale of these plots (scales are chosen to show the more informative, non-zero rates clearly). Metapopulation parameters were a mean dispersal distance of 8 cells, fecundity of 100 and per-cell extinction rate of 0.2. Correlation with speed of advance r = 0.82 for conductivity, r = 0.65 for maximum flow, 0.54 for 1/shortest path and 0.50 for 1/multiple shortest path (variables not log-transformed for correlation calculation). Notice that all metrics predict the large difference between the patchy and channeled types of landscape, but the single and multiple shortest path metrics seriously overestimate the benefit that could be gained by adding a few stepping stones to the patchy landscapes. (PDF) [file pone.0047141.s002.pdf]

Jenny A. Hodgson, Chris D. Thomas, Calvin Dytham, Justin M.J. Travis and Stephen J. Cornell 2012

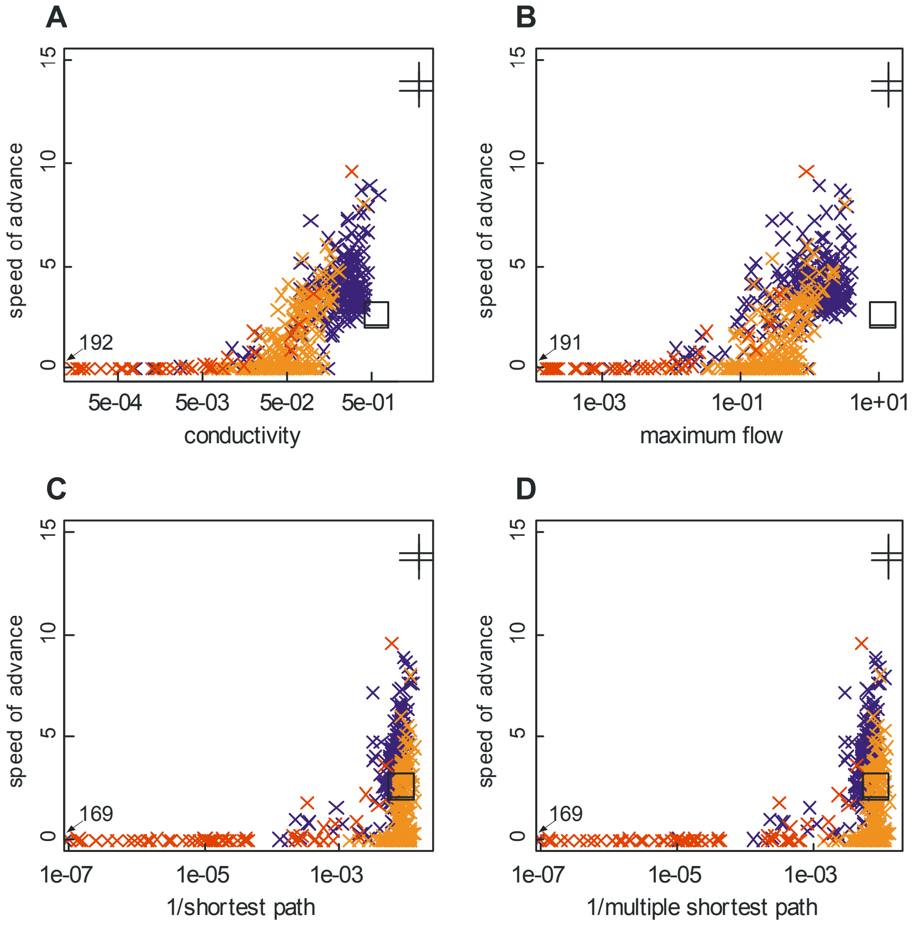

Figure S2: Speed of expansion in simulations versus four putative summary metrics of the landscape. In all panels (a-d) the y axis is the rate of advance (cells/time step) of a simulated metapopulation across one of 332 landscapes (see Fig. 2) in one of 2 directions (east-west or south-north). Each point represents one simulation run. Red points are from patchy landscapes, blue from channeled landscapes and orange from patchy landscapes with stepping stones. Large black cross represents the cross landscape and square represents the regular landscape. The four different metrics (x axes) are explained in methods section "Landscape summary metrics". The numbers at bottom-left indicate the number of points that have a speed indistinguishable from zero and a metric value beyond the scale of these plots (scales are chosen to show the more informative, non-zero rates clearly). Metapopulation parameters were a mean dispersal distance of 8 cells, fecundity of 100 and per-cell extinction rate of 0.2. Correlation with speed of advance r=0.82 for conductivity, r=0.65 for maximum flow, 0.54 for 1/shortest path and 0.50 for 1/multiple shortest path (variables not log-transformed for correlation calculation). Notice that all metrics predict the large difference between the patchy and channeled types of landscape, but the single and multiple shortest path metrics seriously overestimate the benefit that could be gained by adding a few stepping stones to the patchy landscapes.
