# Supplementary material for: The Speed of Range Shifts in Fragmented Landscapes
Source: PLoS One. 2012 Oct 17;7(10):e47141. doi: 10.1371/journal.pone.0047141 (PMC3474837; doi:10.1371/journal.pone.0047141)
Supplement: Figure S3 — Speed of expansion in simulations for species with different dispersal distances. In all panels the y axis is the rate of advance (cells/time step) of a simulated metapopulation across one of 332 landscapes (see Fig. 2) in one of 2 directions (east-west or south-north), and the x axis is the conductivity, whose value depends on the landscape arrangement and the dispersal kernel. Each point represents one simulation run. Red points are from patchy landscapes, blue from channeled landscapes and orange from patchy landscapes with stepping stones. Large black cross represents the cross landscape and square represents the regular landscape. Metapopulation parameters were fecundity R = 100 and per-cell extinction rate μ = 0.2. We observed that rate of advance is approximately equal to conductivity ×√R, plotted as a thick grey line. The black lines show the points where observed/predicted speed would be insufficient for the species to advance across the landscape in the maximum time allowed for the simulation (which was 10,000 time steps for a dispersal distance of 8, 20,000 for 4 and 40,000 for 2), and numbers denote the count of runs falling above or below these lines. Points with conductivity less than 10−4, all with speeds indistinguishable from zero, are not shown on the graph but are included in the counts. (PDF) [file pone.0047141.s003.pdf]

SUPPLEMENTARY MATERIAL FOR “THE SPEED OF RANGE SHIFTS IN FRAGMENTED LANDSCAPES”

Jenny A. Hodgson, Chris D. Thomas, Calvin Dytham, Justin M.J. Travis and Stephen J. Cornell 2012

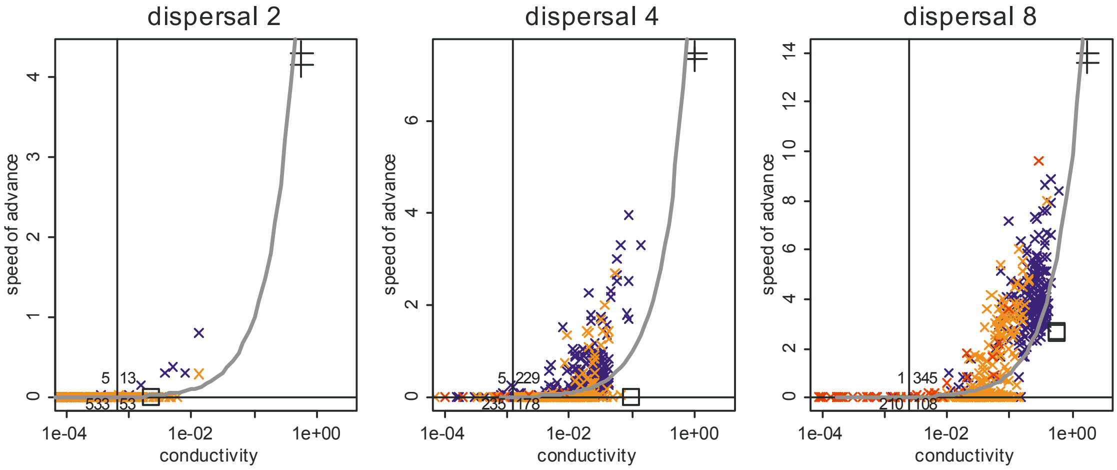

Figure S3: Speed of expansion in simulations for species with different dispersal distances. In all panels the y axis is the rate of advance (cells/time step) of a simulated metapopulation across one of 332 landscapes (see Fig. 2) in one of 2 directions (east-west or south-north), and the x axis is the conductivity, whose value depends on the landscape arrangement and the dispersal kernel. Each point represents one simulation run. Red points are from patchy landscapes, blue from channeled landscapes and orange from patchy landscapes with stepping stones. Large black cross represents the cross landscape and square represents the regular landscape. Metapopulation parameters were fecundity R = 100 and per-cell extinction rate $\mu = 0.2$. We observed that rate of advance is approximately equal to conductivity $\times\sqrt{R}$, plotted as a thick grey line. The black lines show the points where observed/predicted speed would be insufficient for the species to advance across the landscape in the maximum time allowed for the simulation (which was 10,000 time steps for a dispersal distance of 8, 20,000 for 4 and 40,000 for 2), and numbers denote the count of runs falling above or below these lines. Points with conductivity less than 10^{-4}, all with speeds indistinguishable from zero, are not shown on the graph but are included in the counts.
