# Supplementary material for: The Speed of Range Shifts in Fragmented Landscapes
Source: PLoS One. 2012 Oct 17;7(10):e47141. doi: 10.1371/journal.pone.0047141 (PMC3474837; doi:10.1371/journal.pone.0047141)
Supplement: Figure S4 — Speed of expansion in simulations for species with different fecundity and extinction rates (R and μ). In all panels the y axis is the rate of advance (cells/time step) of a simulated metapopulation across one of 332 landscapes (see Fig. 2) in one of 2 directions (east-west or south-north), and the x axis is the conductivity. Each point represents one simulation run. Dispersal distance equals 8 for all panels. We observed that rate of advance is approximately equal to conductivity ×√R, plotted as a thick grey line. The black lines show the points where observed/predicted speed would be insufficient for the species to advance across the landscape in the maximum time allowed for the simulation (200 time steps). Red points are from runs where the starting patch occupancy (at the end of the 200 time-step burn-in) was less than 2/3, to show that these landscapes tended to have a lower speed than predicted from their conductivity. (PDF) [file pone.0047141.s004.pdf]

SUPPLEMENTARY MATERIAL FOR "THE SPEED OF RANGE SHIFTS IN FRAGMENTED LANDSCAPES"

Jenny A. Hodgson, Chris D. Thomas, Calvin Dytham, Justin M.J. Travis and Stephen J. Cornell 2012

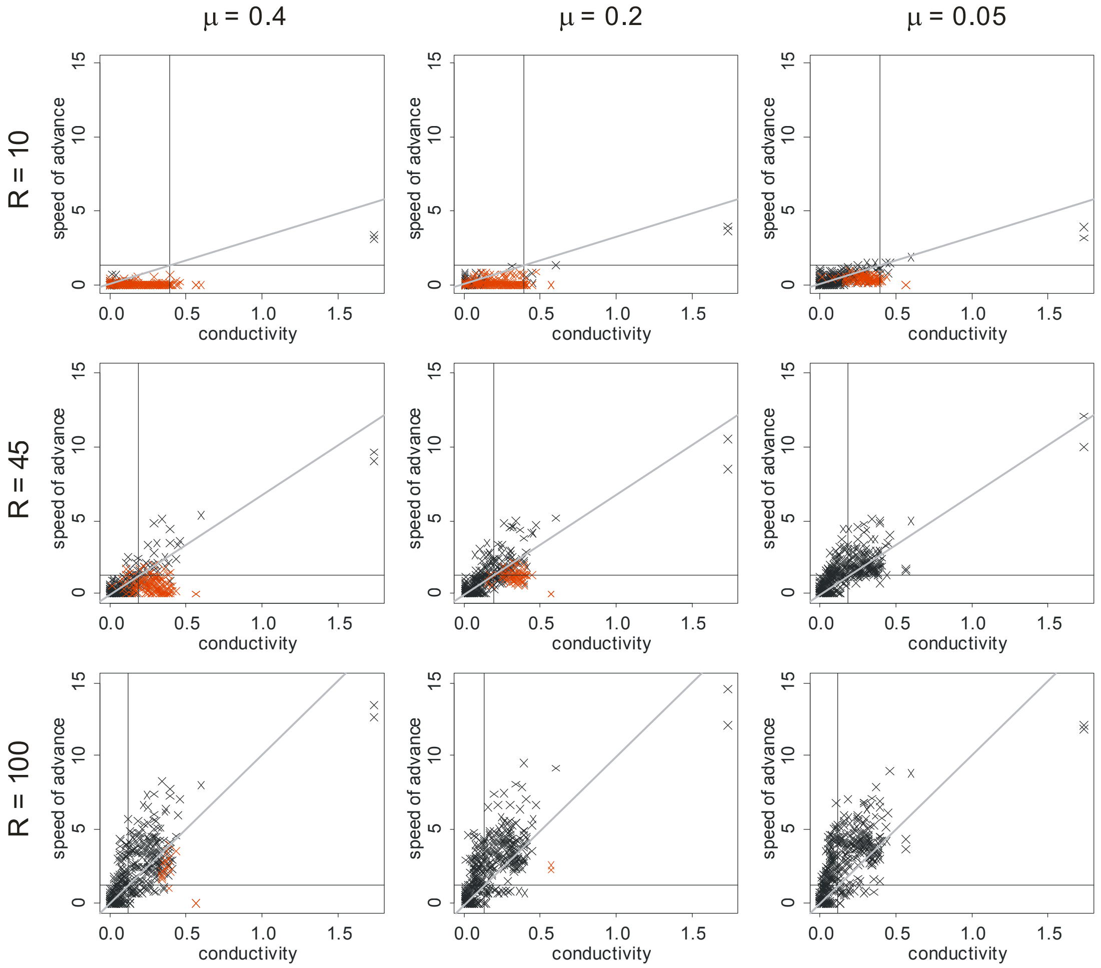

Figure S4: Speed of expansion in simulations for species with different fecundity and extinction rates (R and μ). In all panels the y axis is the rate of advance (cells/time step) of a simulated metapopulation across one of 332 landscapes (see Fig. 2) in one of 2 directions (east-west or south-north), and the x axis is the conductivity. Each point represents one simulation run. Dispersal distance equals 8 for all panels. We observed that rate of advance is approximately equal to conductivity $\times\sqrt{R}$, plotted as a thick grey line. The black lines show the points where observed/predicted speed would be insufficient for the species to advance across the landscape in the maximum time allowed for the simulation (200 time steps). Red points are from runs where the starting patch occupancy (at the end of the 200 time-step burn-in) was less than 2/3, to show that these landscapes tended to have a lower speed than predicted from their conductivity.
